# Hydrolyzed chicken liver used as single source of animal protein in diet and its effect on cytokines, immunoglobulins, and fecal microbiota profile of adult dogs

**DOI:** 10.1371/journal.pone.0271932

**Published:** 2022-07-22

**Authors:** Caroline Fredrich Dourado Pinto, Bianca Brum de Oliveira, Marcelino Bortolo, Ryan Guldenpfennig, Fábio Ritter Marx, Luciano Trevizan

**Affiliations:** 1 Laboratório de Ensino Zootécnico, Animal Science Department, Universidade Federal do Rio Grande do Sul, Porto Alegre, Rio Grande do Sul, Brazil; 2 R&D Manager, Nutrisurance Division, Kemin Industries, Inc., Indaiatuba, São Paulo, Brazil; 3 R&D Scientist, Nutrisurance Division, Kemin Industries, Inc., Des Moines, Iowa, United States America; University of Life Sciences in Lublin, POLAND

## Abstract

Dogs with food allergies and enteropathies may require hydrolyzed diets to prevent or reduce clinical signs, therefore the protein sources used in these diets must be previously characterized and evaluated in healthy dogs. This study aimed to investigate the effects of a hydrolyzed chicken liver powder-based diet (HCLP) versus a poultry by-product meal and bovine meat and bone meal-based diet (Control), on complete blood count (CBC), cytokine, immunoglobulins responses (assessed on days 0, 15, 30 and 45), and fecal microbiota (assessed on day 45) in healthy adult dogs. The CBC did not differ between diets (P>0.05), remaining within reference range. Total plasma IL-4 concentrations were decreased over time independent of the dietary treatment (P<0.001). Total plasma IgA decreased on day 30 compared to days 0 and 45 in dogs fed the control diet (P<0.001). Total plasma IgE concentrations were reduced on days 30 and 45 in dogs fed the control diet, and on days 15 vs 30 and 15 vs 45 in dogs fed HCLP diet (P = 0.001). The 16S rRNA gene sequencing showed similar species richness and abundances of phyla and genera between diets (P>0.05). β-diversity principal coordinate analysis plots demonstrated that HCLP group had a higher similarity than control. Based on our results, healthy adult dogs fed a HCLP based diet maintained normal values for hematological and immunological characteristics, and fecal microbiota after 45 days of feeding.

## Introduction

Hydrolyzed proteins are commonly used in therapeutic diets for dogs with adverse food reactions (AFR) and gastrointestinal diseases. These therapeutic diets possess a high bioavailability of nutrients and peptides with reduced molecular weight that decrease the risk of antigenic stimulation in the intestine [1]. Food antigens elicit an immune response at the Gut Associated Lymphoid Tissue (GALT) after passing through the intestinal mucosa, which can lead to immediate (IgE-mediated, Type I reaction) or delayed (immune complex or cell mediated, also known as Types III and IV, respectively) hypersensitivities. Whether through IgE induced mast cell degranulation or by humoral and cell-mediated reactions, the constant stimulus induces symptoms such as pruritus, vomiting, and diarrhea in dogs [2]. Several researchers reported increased TNF-α and IL-4, and decreased IL-10 in humans and mice with food allergies [3–5]. In dogs, previous studies highlighted the benefits of hydrolyzed proteins in preventing allergenic reactions. Puigdemont et al. [6] observed that experimentally soy-sensitized dogs did not develop cutaneous and gastrointestinal reactions after oral administration of hydrolyzed soy protein. In a different approach, Olivry et al. [7] showed that only an extensively hydrolyzed poultry feather extract was able to prevent recognition by serum IgE from poultry-sensitized dogs.

Since hydrolysis allows the cleavage of the protein chains into small peptides and free amino acids [1], we presumed that the hydrolyzed protein diet could modulate the gut microbiota composition and functionality due to the high bioavailability of peptides present in the chicken liver hydrolyzed protein resulting in the formation of less protein fermentation end products. Bacterial metabolic products synthesized in the colon from “bypass” undigested nutrients [8] generated from proteins can be harmful to the host gastrointestinal health, such as ammonia, sulfides, phenols, and biogenic amines [9, 10].

While hydrolyzed proteins may stimulate less cytokine production than intact proteins, in order to guarantee the safety of novel ingredients it is highly recommended to investigate their effects on healthy dogs. Thus, this study aimed to evaluate the effects of a hydrolyzed chicken liver diet on hematologic, pro-inflammatory (IFN-γ, TNF-α, IL-2, IL-6, IL-7) and anti-inflammatory (IL-4, IL-10) cytokines, immunoglobulins (IgA and IgE), and fecal microbiome response of healthy adult dogs.

## Materials and methods

Animal care and handling procedures were approved by The Institutional Animal Care and Use Committee at the Universidade Federal do Rio Grande do Sul (protocol number 36138).

### Animals and installations

Twelve healthy, intact adult Beagle dogs (6 males and 6 females), 5 years old, weighing 11.8 ± 1.45 kg, body condition score ranging from 5 to 6 out of 9 points [11], and free of ectoparasites were supplied by the Animal Science Department, Universidade Federal do Rio Grande do Sul–UFRGS, Porto Alegre, Brazil. The dogs were regularly immunized, de-wormed, and submitted to clinical and laboratory tests to measure complete blood count and biochemistry analyses before the trial beginning. The dogs were allocated into individual stainless steel metabolic cages (1.0 × 1.0 × 1.5 m) equipped with a feces and urine collector, feeders, and drinkers, in a controlled room at 24°C, with a light: dark cycle of 14:10 h. During the study, they were fed twice daily inside the metabolic cages and stayed there all through the night. During the day dogs remained all together in an outdoor area for socialization. After the study, the dogs remained in the lab for further nutritional studies.

### Experimental design

The study was conducted in a randomized design with two dietary treatments, with six dogs per diet. The experimental period lasted 45 days in which was measured: hematological indicators, cytokines (IFN-γ, TNF-α, IL-2, IL-4, IL-6, IL-7, IL-10) and immunoglobulins (IgA and IgE) on days 0, 15, 30 and 45; and fecal microbiota on day 45.

### Dietary treatments

Before the start of the study, all dogs were fed the same commercial diet for 30 days, during a wash-in period to stabilize the gastrointestinal tracts. Experimental diets ingredients and chemical composition were previously reported by Pinto et al. [12] and are presented in Table 1. The dietary treatments were a Control diet (based in poultry by-product + bovine meat and bone meals) and HCLP diet (based in hydrolyzed chicken liver powder—PROSURANCE® CHX Liver HD, Kemin® Industries). The dogs received the dietary treatments twice a day (at 08:30 h and 17:00 h) to attend their daily maintenance energy requirements (110 kcal of metabolizable energy x body weight (kg)^0.75^/day), as recommended by NRC [13]. The dogs were weighed weekly to adjust the amount of food and maintain a body condition score ranging from 5 to 6 out of 9 points. Water was provided *ad libitum* during the trial.

**Table 1:** Ingredients and chemical composition of experimental diets.

Ingredient, % as fed basis	Treatments	
	Control	HCLP
Brewers rice	52.7	52.7
Full-fat rice bran	8.00	8.00
Poultry by-product meal	18.6	-
Bovine meat and bone meal	5.00	-
HCLP^1^	-	25.8
Cellulose	3.34	4.22
Poultry fat^2^	8.75	4.30
Soybean oil^2^	-	1.80
Canola oil^2^	1.37	0.46
L-lysine	0.63	-
DL-methionine	0.33	0.27
Potassium chloride	0.23	0.07
L-tryptophan	0.03	-
Limestone	-	2.01
Premix mineral/vitamin^3^	0.50	0.50
Salt	0.50	0.50
Analyzed composition, % DM basis		
Dry matter	94.1	94.5
Organic matter	94.3	95.4
Ash	5.67	4.62
Crude protein	24.8	24.8
Acid-hydrolysed fat	15.1	14.0
Crude fiber	3.75	4.67
Gross energy, kcal/kg	4950	5055

Control, poultry by-product + bovine meat and bone meals-based diet; HCLP, hydrolyzed chicken liver powder-based diet.

^1^HCLP: hydrolysed chicken liver powder ingredient.

^2^Added on top.

^3^Premix mineral/vitamin (supplied per kilogram of diet): vitamin A (10,800U), vitamin D3 (980 U), vitamin E (60 mg), vitamin K3 (4.8 mg), vitamin B1 (8.1 mg), vitamin B2(6.0 mg), vitamin B6 (6.0 mg), 12 vitamin (30 mcg), pantothenic acid(12 mg), niacin (60 mg), folic acid (0.8 mg), biotin (0.084 mg), manganese(7.5 mg), zinc (100 mg), iron (35 mg), copper (7.0 mg), cobalt (10 mg), iodine (1.5 mg), selenium (0.36 mg), choline (2.400 mg), taurine (100 mg),and, antioxidant BHT (150 mg).

### Immunological analyses

To assess the effect of a hydrolyzed chicken liver diet on systemic humoral responses, circulating total IFN-γ, TNF-α, IL-2, IL-4, IL-6, IL-7, IL-10, IgA, and IgE were measured in the plasma.

#### Sample collection

Blood samples were collected via jugular venipuncture before morning feeding sessions on days 0, 15, 30, and 45 of the experimental periods. Three mL blood samples were placed into blood collection tubes with EDTA per dog on each time of the evaluation. One tube was used for complete blood count (CBC). The other two tubes were centrifuged at 1,000 *g* for 15 min within 30 minutes after blood collection. Then the plasma was immediately divided into aliquots, transferred to 2 mL Eppendorf tubes, and stored frozen at -20°C until cytokine and immunoglobulin analyses were performed.

#### Cytokines and immunoglobulins analyses

IFN-γ, TNF-α, IL-2, IL-6, IL-7, and IL-10 were quantified using a Luminex xMAP kit canine specific, according to the manufacturer’s instructions (CCYTOMAG-90K - MILLIPLEX MAP Canine Cytokine Panel). The presence of IL-4 was quantified using an ELISA kit specific to dogs, according to the manufacturer’s instructions (Canine IL-4 ELISA, SEA077CA). The plasmatic levels of immunoglobulins, IgA (IgA Dog ELISA ab157699, Abcam®) and IgE (IgE Dog ELISA ab157700, Abcam®) were performed using ELISA kits specific to dogs, used according to the manufacturer’s instructions.

### Fecal microbiota analyses

Fecal samples from each dog were collected the morning of day 45. Samples were divided into aliquots, stored in a 2 mL Eppendorf tube, and stored in a freezer at -20°C until analyzed. Total DNA was extracted using ZR Fecal DNA MiniPrep (Zymo Research Corporation, Irvine, CA), followed by quantification of extracted DNA by spectrophotometry at 260nm. Quality of extracted DNA was assessed by electrophoresis using a 1% agarose gel. A segment of approximately 460 bases from the hypervariable region V3V4 of the ribosomal 16S rRNA gene was amplified, under the following PCR conditions: 95°C for 3 min; 25 cycles of 95°C for 30 sec, 55°C for 30 sec and 72°C for 30 sec, followed by step at 72°C for 5 min. The metagenomic library was built from these amplifiers using the commercial kit Nextera DNA Library Preparation Kit (Illumina Inc., San Diego, CA). Amplicons were pooled and sequenced using the Illumina MiSeq sequencer (Illumina Inc., San Diego, CA) [14]. Sequence data was analyzed using the QIIME (Quantitative Insights Into Microbial Ecology) platform [15, 16], followed by a low quality sequence removal workflow, filtration, chimera removal and taxonomic classification. Sequences were classified into bacterial genera through the recognition of operational taxonomic units (OTU), in this case, the homology between the sequences when compared to a database. Sequences were compared using the update 2017 version (SILVA 128) from the database of ribosomal RNA genes sequences SILVA [17]. In total, 63000 readings were used per sample to generate the classification of bacterial communities through the identification of OTU, aiming to normalize the data and not compare samples with different numbers of readings, avoiding taxonomic bias. Alpha diversity was estimated using OTU, Simpson Index, and Shannon Index. Beta diversity was calculated using Bray-Curtis and D_0.5 UniFrac distance measures and presented with Principal Coordinate Analysis (PCoA) plots.

### Statistical analysis

Complete blood count, cytokines and immunoglobulins were analyzed as delta values, calculated by the difference between each evaluated day and day 0. A positive delta represents an increase in the measure while a negative delta represents a reduction in time. All data were analyzed using the GEE procedure (Generalized Estimating Equation) with the Bonferroni post-hoc test for days. Data without normal distribution were previously transformed and analyzed with gamma distribution for transformed variables. Analyses were performed using the software IBM SPPS v. 18 (Statistical Package for Social Sciences) and the level of 5% was considered significant. Additionally, hematological and immunological markers were submitted to a Multivariate Analysis of Variance (MANOVA) and Canonical Discriminant Analysis to verify the differences and associations between the dietary treatments throughout the experimental period, also considering the level of 5% as significant. Alpha diversity was estimated by Kruskal-Wallis H-test. Differences between the relative abundances of the taxonomic groups were estimated by T-test using the software Minitab 18 (Minitab Inc. State College, PA) and the level of 5% was considered significant.

## Results

All hematological indicators were within the reference values. There was an increase over time independent of the dietary treatment for erythrocytes (P = 0.0110) and hemoglobin on day 15 (P = 0.0010), hematocrit on days 15 and 45 vs day 0 (P<0.001) and mean corpuscular volume on days 15 and 45 vs day 0 (P<0.001) (Table 2). A reduction over time regardless of dietary treatment was detected in mean corpuscular hemoglobin (P = 0.0360), leucocytes on days 15 and 30 vs day 0 (P<0.001), total segmented neutrophils on day 30 vs day 0 (P<0.001), total monocytes on days 15 vs 0 and 15 vs 30 (P<0.001). Changes over time were also observed for total lymphocytes on days 30 vs 45 (P = 0.0140), monocytes on days 15 vs 0 and days 15 vs 30 (P<0.001), and lymphocytes on days 30 and 45 vs 0 (P = 0.0010). Interaction effect was observed for total eosinophils, in which the HCLP group had a difference on day 45 (P = 0.040). Eosinophils had a difference in the control group on days 15 vs 30, and HCLP group on days 45 vs 0, and 45 vs 30 (P = 0.0280). Total protein differed on the control group on days 15 vs 30, and on HCLP group on days 0 vs 15 and 45, 15 vs 30, 30 vs 45 (P = 0.008).

**Table 2:** Hematology of dogs fed the experimental diets.

Items	RV	Treatments^1^				Δ-values^2^			P-values		
		D0	D15	D30	D45	D 15–0	D 30–0	D 45–0	Diet	Day	Diet x Day
Erythrocytes, 106/μL^3^											
Control	5.5–8.5	6.64±0.19	6.74±0.09	6.79±0.09	6.94±0.06	0.10	0.15	0.30	0.251	0.011	0.145
HCLP		6.63±0.27	7.11±0.11	6.97±0.09	7.21±0.04	0.48	0.34	0.59			
Hemoglobin, g/dL^3^											
Control	12–18	15.4±0.43	15.8±0.18	15.8±0.16	16.1±0.10	0.40	0.43	0.75	0.441	0.001	0.083
HCLP		15.4±0.55	16.6±0.20	16.0±0.17	16.7±0.07	1.15	0.58	1.25			
Hematocrit, %^3^											
Control	37–55	44.7±1.22	46.0±0.49	46.0±0.32	47.5±0.24	1.33	1.33	2.83	0.405	<0.001	0.263
HCLP		44.5±1.56	48.0±0.40	46.7±0.43	48.8±0.20	3.50	2.17	4.3			
MCV, fL^3^											
Control	60–77	67.3±0.71	68.5±0.32	67.9±0.41	68.5±0.22	1.15	0.58	1.22	0.391	<0.001	0.638
HCLP		67.3±0.40	67.7±0.31	67.1±0.34	67.8±0.32	0.35	-0.22	0.43			
MCHC, %^3^											
Control	32–36	34.5±0.18	34.3±0.08	34.4±0.13	34.0±0.05	-0.15	-0.07	-0.50	0.847	0.036	0.8177
HCLP		34.6±0.15	34.5±0.09	34.3±0.06	34.2±0.06	-0.13	-0.33	-0.48			
Leucocytes, /μL^4^											
Control	6000–17000	10167±367	9417±240	9133±189	8733±215	-400	-800	-1150	0.743	<0.001	0.415
HCLP		9717±508	8367±272	8017±192	8917±422	-1850	-2050	-1350			
Total segmented neutrophils, /μL^4^											
Control	3000–11500	6489±263	5787±250	5280±174	5395±188	-937	-1196	-995	0.910	<0.001	0.724
HCLP		6480±363	5384±168	4922±87.3	6249±388	-1276	-1652	-619			
Total eosinophils, /μL^4^											
Control	100–1250	828±128	678±39.6	477±29.2	516±27.4	-66.5^Aa^	-291^Aa^	-200^Aa^	0.893	<0.001	0.040
HCLP		706±88.3	526±38.9	541±42.2	413±14.6	-217^Ab^	-183^Ab^	-382^Aa^			
Total monocytes, /μL^4^											
Control	150–1350	583.5±62.5	381±24.5	589±43.5	455±23.9	-253	-80.5	-212	0.844	<0.001	0.582
HCLP		636±76.1	398±36.9	599±28.7	660±52.2	-336	-6.00	168			
Total lymphocytes, /μL^3^											
Control	1000–4800	2266±213	2619±96.8	2787±98.2	2368±98.5	353	521	102	0.322	0.014	0.586
HCLP		1895±259	2113±141	1955±103	1706±76.2	218	59.3	-189			
Segmented neutrophils, %^3^											
Control	60–77	64.2±2.54	61.0±1.22	57.7±1.33	61.5±1.57	-3.17	-6.50	-2.67	0.567	0.073	0.480
HCLP		66.8±2.93	65.0±1.76	62.2±1.57	68.8±1.63	-1.83	-4.67	2.00			
Eosinophils, %^3^											
Control	2–10	8.00±0.95	7.17±0.33	5.17±0.33	6.00±0.36	-0.83^Ab^	-2.83^Aa^	-2.00^Aab^	0.915	0.002	0.028
HCLP		7.17±0.64	6.17±0.34	6.50±0.44	5.00±0.44	-1.00^Aab^	-0.67^Ab^	-3.50^Aa^			
Monocytes, %^3^											
Control	3–10	5.67±0.45	4.20±0.30	6.50±0.45	5.17±0.25	-2.00	0.83	-0.50	0.838	<0.001	0.866
HCLP		6.67±0.91	4.80±0.45	7.50±0.31	7.33±0.44	-2.50	0.83	0.67			
Lymphocytes, %^3^											
Control	12–30	22.2±1.68	28.2±1.03	30.7±1.04	27.3±1.20	6.00	8.50	5.17	0.303	0.001	0.169
HCLP		19.3±2.18	24.7±1.20	23.8±0.98	20.2±1.10	5.33	4.50	0.83			
Total protein, g/L^4^											
Control	60–80	54.0±7.35	65.7±0.80	62.7±0.54	55.3±3.43	0.00^Aab^	-2.00^Ab^	1.00^Aa^	0.026	<0.001	0.008
HCLP		62.3±1.54	66.7±0.74	61.0±0.43	68.7±0.74	4.00^Ba^	-1.00^Ab^	5.00^Aa^			
Platelet count, /μL^4^											
Control	200000–500000	366667±14062	3733333±5274	340000±7912	354000±3871	0.00	-15000	-37000	0.103	0.069	0.357
HCLP		309500±30074	389167±18410	331333±8277	315000±5696	48000	-12000	-20000			

RV, reference values; Control, poultry by-product + bovine meat and bone meals-based diet; HCLP, hydrolyzed chicken liver powder-based diet; MCV, mean corpuscular volume; MCHC, mean corpuscular hemoglobin concentration.

^1^Values expressed as mean ± standard error of the mean.

^2^Values expressed as delta values.

^3^GEE (Generalized Estimating Equations) analysis with normal distribution.

^4^GEE (Generalized Estimating Equations) analysis with gamma distribution for transformed variables.

^a,b^Comparisons of days in each treatment in the same row with different lowercase letters are significantly different by Bonferroni post-hoc test (P<0.05).

^A,B^Comparisons of treatments on each day in the same row with different capital letters are significantly different by Bonferroni post-hoc test (P<0.05).

**Fig 1:**
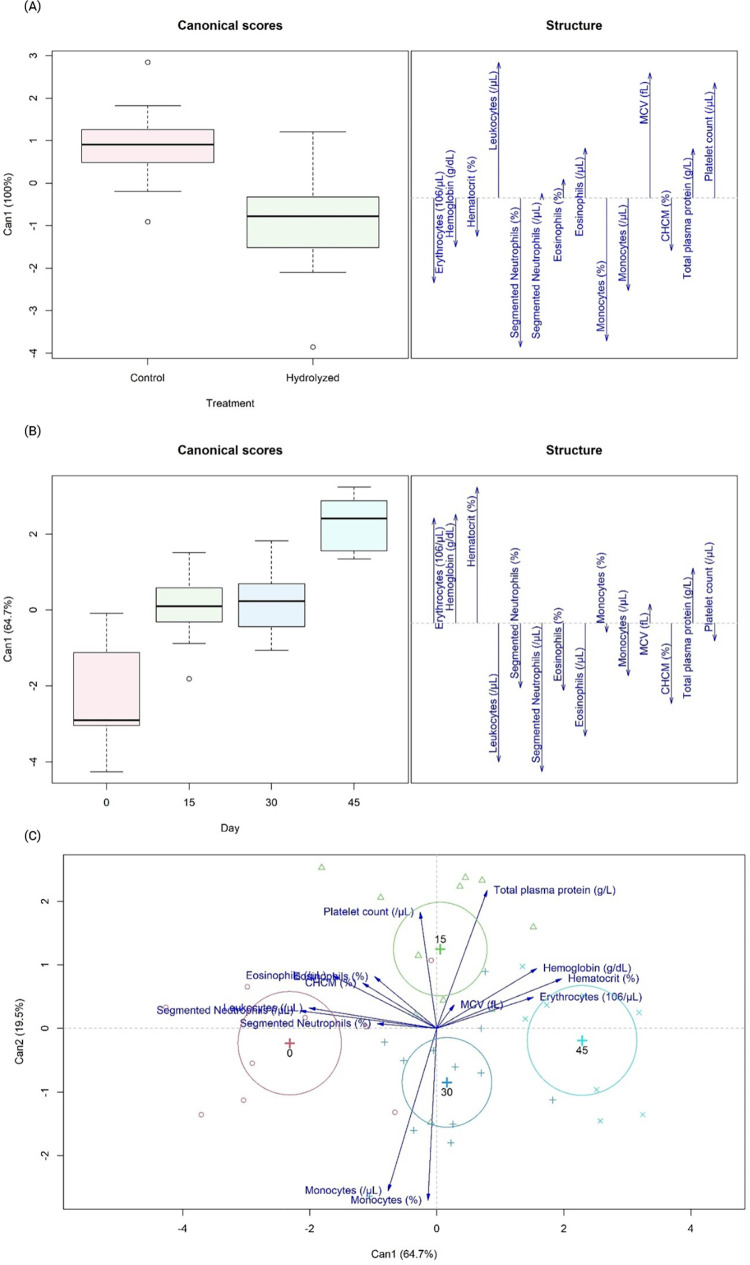
Canonical correlations of dietary treatments (A), days of blood collection (B) and two-dimensional canonical correlation of days of blood collection (C) for hematological markers from healthy Beagle dogs fed the experimental diets.

Although MANOVA did not identify differences between dietary treatments and blood collection days (P>0.05), the canonical correlation identified differences between dietary treatments regarding hematological markers (Fig 1A), in which leukocytes, segmented neutrophils (/μL), eosinophils, MCV, total protein and platelet counts were associated with the control diet, while erythrocytes, hemoglobin, hematocrit, segmented neutrophils (%), monocytes and MCHC were associated with the hydrolyzed diet. Considering the day of blood collection (Fig 1B), the medians of all markers on day 0 were smaller than on other days, but with greater variability. Multivariate analysis did not identify a difference between the days of blood collection, but averages appeared to be greater on day 45, especially when compared to day 0. Through the two-dimensional correlation, it is possible to assess that leukocytes and segmented neutrophils are associated with day 0, while platelet count and total protein are with day 15. Monocytes point to day 30, while hemoglobin, hematocrit, and erythrocytes appear to be associated with day 45 (Fig 1C).

No changes were observed in the total plasma concentrations of IFN-γ, TNF-α, IL-2, and IL-10 during the experimental period (P>0.05) (Table 3). Total plasma IL-4 decreased over time regardless of dietary treatment (P<0.001), with an increase in delta values on days 30 (P = 0.006) and 45 (P<0.001) compared to day 0. In addition, there was a significant difference between the delta values of days 15 and 30 (P = 0.002), and delta values of days 15 and 45 (P<0.001). Although there was a difference between the delta values over time in the total plasma concentrations of IL-6 (P = 0.015) and IL-7 (P = 0.036), it was not possible to verify differences with the Bonferroni post-hoc test. There were differences over time for both dietary treatments in IgA and IgE concentrations (P<0.05). Total plasma IgA delta values differed on day 30 compared to days 0 and 45 for the control group (P<0.001). However, the delta values of the HCLP group showed no differences. The delta values of total plasma IgE decreased for both treatments over time, in the control group the delta values on days 30 and 45 differed from the others, while for the HCLP group the delta values differed on days 15 vs 30 and 15 vs 45 (P = 0.001).

**Table 3:** Plasma cytokines and immunoglobulins concentrations of dogs fed the experimental diets.

Items	Treatments^1^				Δ-values^2^			P-values		
	D0	D15	D30	D45	D 15–0	D 30–0	D 45–0	Diet	Day	Diet x Day
IFN-γ, pg/mL^3^										
Control	1.37±0.43	1.27±0.41	1.33±0.44	1.24±0.37	-0.11	-0.04	-0.13	0.900	0.388	0.354
HCLP	0.88±0.13	0.83±0.11	0.79±0.07	0.79±0.12	-0.05	-0.09	-0.09			
TNF-α, pg/mL^4^										
Control	21.0±7.65	14.4±3.70	10.0±2.18	10.3±2.48	-0.05	-0.73	0.04	0.249	0.225	0.118
HCLP	4.07±0.51	3.31±0.32	3.46±0.38	3.16±0.29	-0.75	-0.68	-0.92			
IL-2, pg/mL^4^										
Control	44.1±15.8	31.2±9.70	27.8±8.27	28.1±9.03	-0.68	-4.27	-0.07	0.163	0.342	0.381
HCLP	7.24±1.25	6.44±1.08	6.99±1.51	5.94±0.85	-0.54	-0.50	-1.25			
IL-4, pg/mL^4^										
Control	174±20.1	163±9.82	125±14.4	115±14.9	-5.22	-42.6	-53.1	0.166	<0.001	0.107
HCLP	178±20.1	174±11.3	157±7.22	142±12.0	-12.8	-29.4	-41.2			
IL-6, pg/mL^4^										
Control	19.9±6.42	15.6±4.70	13.2±3.75	14.7±3.64	-0.64	-3.73	-0.06	0.502	0.015	0.074
HCLP	13.4±5.07	12.3±4.07	11.2±3.19	10.9±3.38	-0.66	-0.11	-1.15			
IL-7, pg/mL^4^										
Control	90.3±34.8	61.3±17.8	42.9±11.1	45.7±12.6	-3.37	-10.6	-2.95	0.160	0.036	0.304
HCLP	21.2±6.14	20.0±5.34	17.3±4.17	11.6±2.09	-0.73	-1.40	-5.43			
IL-10, pg/mL^4^										
Control	6.30±1.42	6.13±1.00	6.25±1.04	7.91±1.57	-0.09	0.00	0.43	0.293	0.168	0.367
HCLP	5.57±0.43	5.26±0.54	6.05±0.69	4.90±0.30	-0.13	0.33	-0.67			
IgA, ng/mL^4^										
Control	3535419±152407	3568147±78565	3474936±100862	3524653±97926	-13702^Aab^	-68848^Ab^	-5322^Aa^	0.047	0.032	<0.001
HCLP	3512716±147054	3674435±34772	3688204±26921	3560697±61867	14990^Aa^	43240^Aa^	-12102^Aa^			
IgE, ng/mL^4^										
Control	249910±26681	235248±16910	192412±14109	203169±15047	-4825Ac	-57157Aa	-47380Ab	0.619	<0.001	0.001
HCLP	276237±45083	261632±26731	243326±25138	232666±25006	-2200Ab	-18840Aa	-26866Aa			

Control, poultry by-product + bovine meat and bone meals-based diet; HCLP, hydrolyzed chicken liver powder-based diet.

^1^Values expressed as mean ± standard of the error of the mean.

^2^Values expressed as delta values.

^3^GEE (Generalized Estimating Equations) analysis with normal distribution.

^4^GEE (Generalized Estimating Equations) analysis with gamma distribution for transformed variables.

^a,b.c^Comparisons of days in each treatment in the same row with different lowercase letters are significantly different by Bonferroni post-hoc test (P<0.05).

^A,B^Comparisons of treatments on each day in the same row with different capital letters are significantly different by Bonferroni post-hoc test (P<0.05).

**Fig 2:**
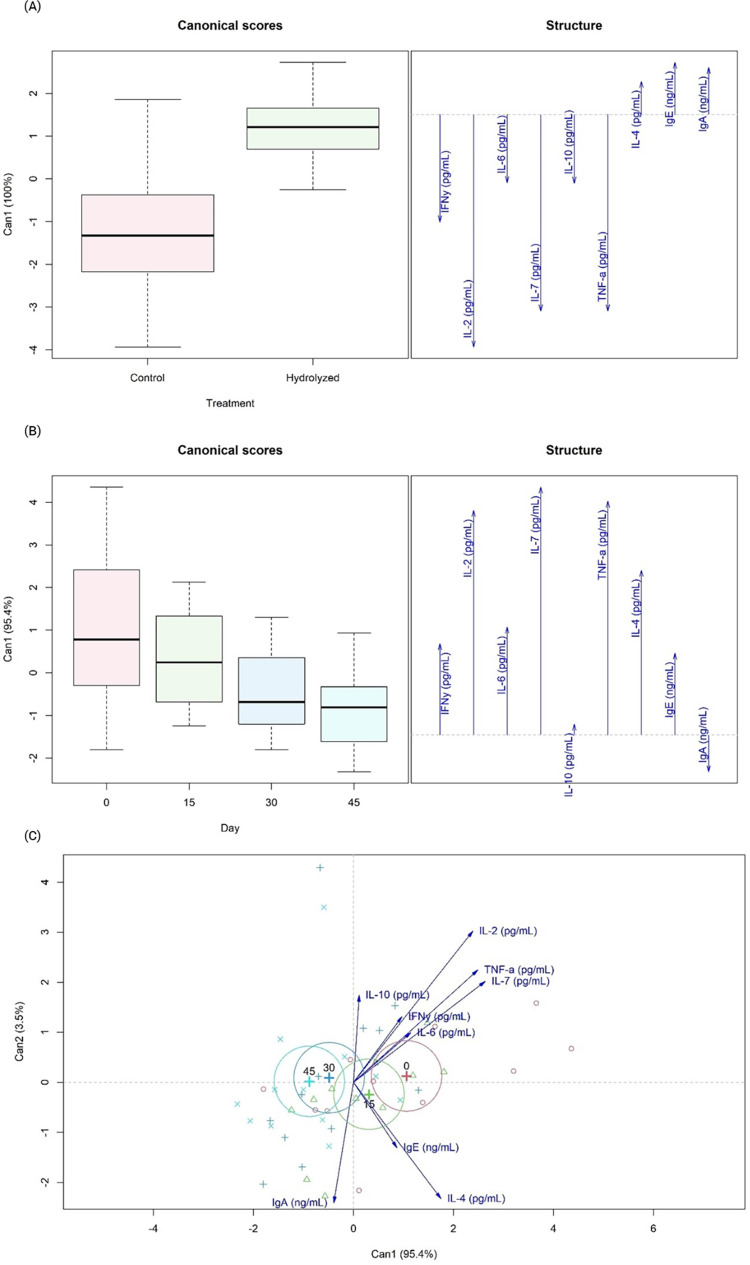
Canonical correlation of dietary treatments (A), days of blood collection (B) and two-dimensional canonical correlation of days of blood collection (C) for plasmatic cytokines and immunoglobulins from healthy Beagle dogs fed the experimental diets.

MANOVA indicated a difference between dietary treatments (P<0.05), but not for the days of blood collection (P = 0.45). The canonical correlation showed the difference between the control and hydrolyzed treatment (Fig 2A), in which markers such as IFN-γ, IL-2, IL-6, IL-7, IL-10, and TNF-α were associated with the control diet, while IL-4, IgE and IgA were associated with the hydrolyzed diet. Regarding the association between immunological markers and days of blood collection (Fig 2B), it seems that the medians were progressively reduced throughout the experimental period, but no statistical difference was observed (P>0.05). The two-dimensional canonical correlation evidenced the difference between day 0 and days 30 and 45 since the circles did not overlap between these days (Fig 2C). In addition, from the attribution of colors, day 30 and day 45 were considered equal.

**Fig 3:**
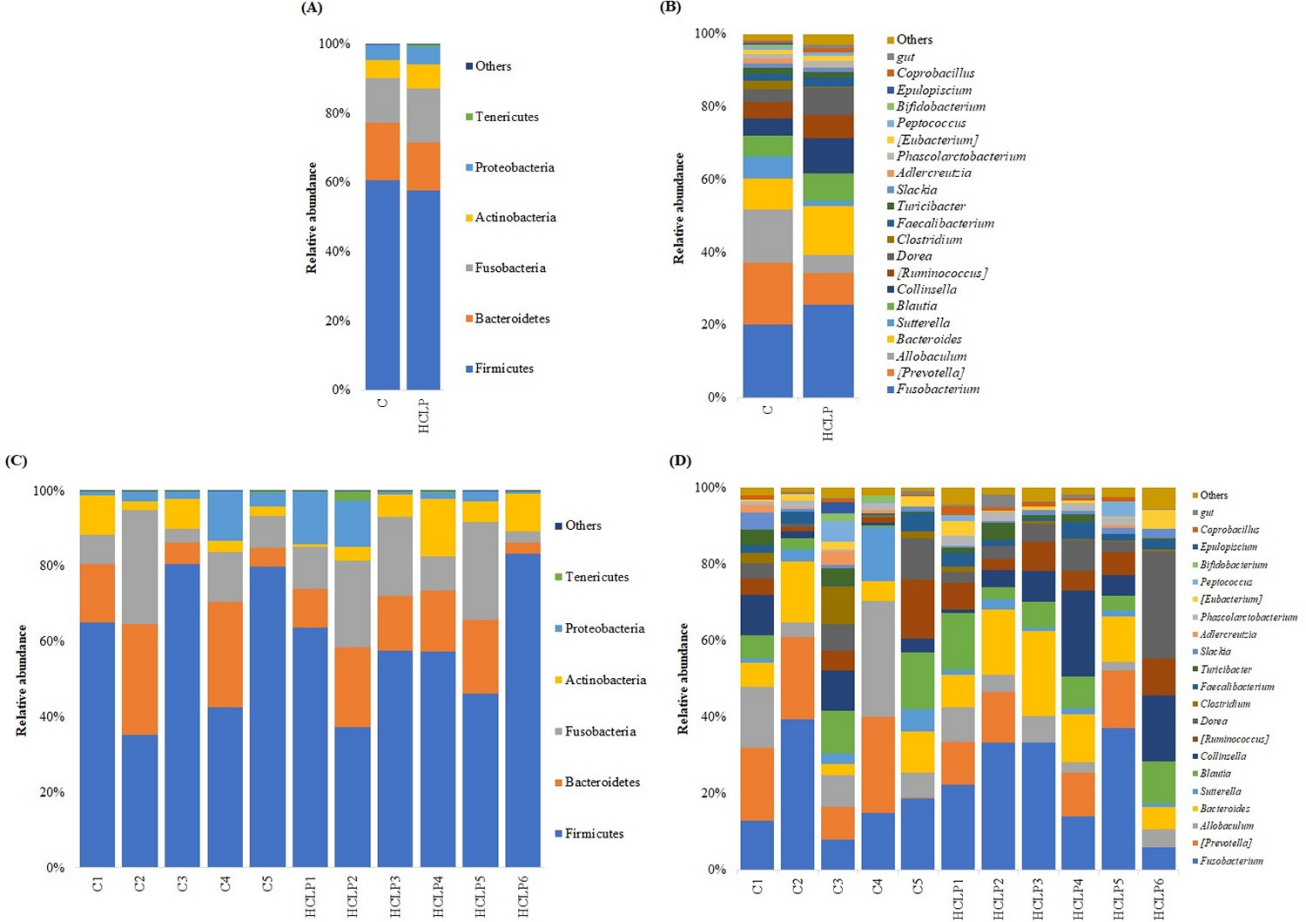
Relative abundances of phylum (A for treatments and C for individuals) and genera (B for treatments and D for individuals) based on 16S sequencing analysis of fecal samples from healthy Beagle dogs after 45 days consuming the experimental diets. Results are displayed for 11 dogs, as 1 dog on the control diet was removed due to the low number of reads. Others: Bacteria taxa with < 1% abundance. C, poultry by-product + bovine meat and bone meals-based diet; HCLP, hydrolyzed chicken liver powder-based diet.

In this study, 11 fecal samples were processed and a total of 63000 sequence reads per dog were used. One fecal sample from the control group was removed due to the low number of read sequences. Bacterial community was composed of 27 phyla, in which 6 phyla (Firmicutes, Bacteroidetes, Fusobacteria, Actinobacteria, Proteobacteria, Tenericutes) represented more than 99% of sequence reads, considering mean relative abundance higher than 0.5%. There was no difference for the relative abundance of phyla between dietary treatments (P>0.05). Both treatments had the majority of bacterial community composed by Firmicutes, Bacteroidetes and Fusobacteria, counting for more than 85% of the OTU (Fig 3A). At the genera level, 152 were identified with 19 of those with a mean relative abundance higher than 0.5%. No changes were observed in the genus abundance between the dietary treatments (P>0.05). The most abundant genera in both treatments were *Fusobacterium*, [*Prevotella*], *Allobaculum*, *Bacteroides*, *Sutterella*, *Blautia*, *Collinsella*, [*Ruminococcus*], *Dorea*, *Clostridium* and *Faecalibacterium*, which counted for more than 50% of the OTU (Fig 3B). Each dog was considered an experimental unit, totaling 6 replicates for the HCLP group and 5 replicates for the control group. Clear differences on abundances of phyla (Fig 3C), but mainly at the genera level (Fig 3D), were detected among dogs within the same dietary treatment on day 45.

**Fig 4:**
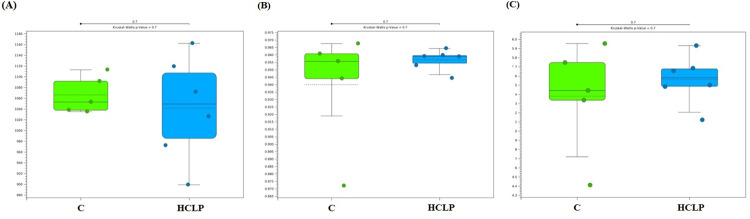
Bacterial alpha diversity indices of canine fecal samples assessed by the observed operational taxonomic units (OTU) (A), Simpson Index (B) and Shannon Index (C). C, poultry by-product + bovine meat and bone meals-based diet; HCLP, hydrolyzed chicken liver powder-based diet.

**Fig 5:**
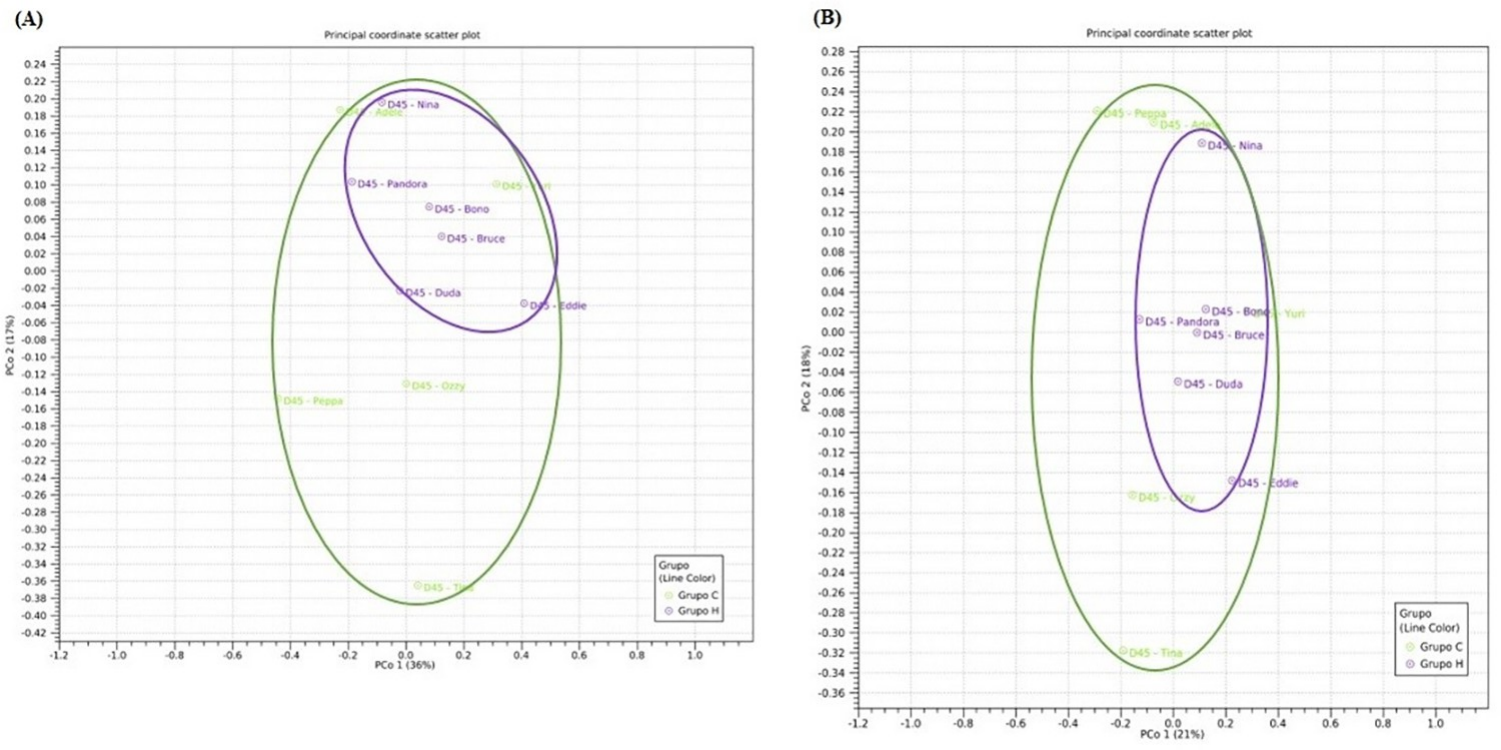
Principal Coordinate Analysis (PCoA) plots of Bray-Curtis (A) and D_0.5 UniFrac (B) distances of fecal samples. Treatments are represented as: Control in green, and HCLP in purple. Control, poultry by-product + bovine meat and bone meals-based diet; HCLP, hydrolyzed chicken liver powder-based diet.

Diversity indices estimators OTU (mean ± SD; HCLP = 1042 ± 96.8; Control = 1066 ± 34.6) (Fig 4A), Simpson Index (HCLP = 0.96 ± 0.007; Control = 0.94 ± 0.039) (Fig 4B) and Shannon Index (HCLP = 5.56 ± 0.27; Control = 5.38 ± 0.59) (Fig 4C) were not affected by the dietary treatments (P = 0.70). Principal Coordinate Analysis (PCoA) based on the Bray-Curtis and D_0 UniFrac distance metrics, the HCLP dogs presented a clustering effect demonstrating a higher similarity degree (Fig 5A and 5B, respectively) differently from those belonging to the control group.

## Discussion

In this study, we replaced the traditional sources of animal protein used in pet food, poultry by-product and bovine meat and bone meals, with a source of hydrolyzed chicken liver powder and observed the effects on CBC, immunological, and fecal microbiota of healthy dogs. Since dietary management of dogs diagnosed with AFR and chronic enteropathies generally includes hypoallergenic diets based on low-molecular weight hydrolyzed proteins to reduce intestinal antigenic stimulation [1, 18], we hypothesized that healthy, immunotolerant dogs fed a hydrolyzed chicken liver diet would not develop strong disturbances in the markers assessed during a period of 45 days.

Most hematological biomarkers were modified throughout the study, but all remained within the reference ranges for healthy adult dogs [19] and the colony norms. According to Sasaki et al. [20], the production of erythropoietin increases as the amount of protein in the diet increases. Based on this, we assume that both diets provided adequate amounts of protein, allowing an increase in erythropoiesis and, consequently, in the erythrocyte, hemoglobin and hematocrit count, verified in both treatments over time. Although there were no significant differences in these markers between dietary treatments, it was possible to observe a greater increase from day 15 onwards in dogs fed the HCLP diet.

Eosinophil counts were affected by both dietary treatments and time, with a progressive reduction throughout the study. However, the HCLP diet promoted a greater and sustained reduction after 45 days of consumption. The increased concentration of eosinophils reflects an immune response to parasites and more recently with some specific cases of diet-related gastrointestinal disorders in dogs [21, 22]. Animals with chronic enteropathies may respond to a single dietary management based on the use of single novel protein or hydrolyzed protein diets, or to a combination of dietary intervention with antibiotics or immunosuppressive agents [23]. The reduction in eosinophil count in dogs fed HCLP diet at day 45 suggests that a beneficial impact on CBC is occurring. As eosinophils are related to the allergic process, the reduction in their counts may be related to the positive impact of the diet. Furthermore, other biomarkers related to immune responses such as leukocytes, neutrophils, and monocytes, were decreased over time in both dietary treatments, which may corroborate the positive impact of HCLP diet consumption in the long term.

Regarding the immunological response, all dogs had been naturally sensitized to poultry by-product meal and bovine meat and bone meal prior to this study, as both ingredients are commonly present in commercial diets for dogs. However, none of them consumed HCLP previously, so if a dog developed a response to HCLP it would be related mainly to this ingredient.

Anti-inflammatory Th2 cytokine, IL-4, was reduced from day 15 to the end of the experimental period in both treatments. IL-4 is one of the signature cytokines involved in inflammatory responses triggered by allergens or parasites [24]. The presence of food allergens in the intestinal mucosa activates antigen-presenting cells (APC) that signal Th2 lymphocytes to secrete cytokines, including IL-4, which stimulate the production of IgE. In addition, APC secrete pro-inflammatory mediators that induce epithelial cells to produce chemokines that attract eosinophils [25]. Thus, the reduction in IL-4 over 45 days in this study may be correlated with the decrease in eosinophil and IgE concentrations. Interestingly, previous studies reported an increase in IL-4 concentrations in humans and mice affected by food-induced gastrointestinal disorders [4, 5]. Additionally, recent reports did not find detectable amounts of IL-4 using ex-vivo whole blood stimulation with commercial hydrolyzed protein diets in dogs with chronic enteropathy and healthy immunotolerant cats [26, 27].

Although plasma IgA was reduced on day 30 in dogs fed the control diet, at the end of the experimental period there was no difference between groups. Similarly, Verlinden et al. [2] also did not observe differences on total serum IgA between dogs fed diets containing hydrolyzed or intact protein. Indeed, some researchers demonstrated that serum IgA concentrations poorly correlates with secretory IgA, the most predominant secretory antibody at intestinal mucosa that protect against microorganisms, allergens, and other substances to adhere and pass-through enterocytes [28, 29]. In this way, future studies should measure secretory IgA to provide a better understanding between dietary treatments and their interference in the immune status of the intestinal mucosa.

IgE concentrations were measured as IgE-mediated reactions are the most recognized category of food allergies. Total plasma IgE concentrations were decreased from day 30 onwards in the control group, while the reduction in the HCLP group was observed from day 15 onwards. Consumption of hydrolyzed diets, containing smaller peptides, rich in free amino acids and low-molecular weight proteins, may reduce the cross binding between two molecules on the surface of the mast cell, thus preventing mast cell degranulation and cytokines release [30]. Verlinden et al. [2] did not observe differences on total serum IgE concentrations of healthy adult dogs fed hydrolyzed protein or intact protein diets. The authors evaluated the total serum IgE only after 24 days of feeding and did not analyze levels at the beginning of the trial, which may have indicated possible changes in this immunoglobulin during the study. Apart from healthy individuals, previous studies on sensitized dogs challenged with hydrolyzed protein diets have shown positive results. Soy-sensitized dogs did not present cutaneous or gastrointestinal reactions after being fed with hydrolyzed soy protein diet [6]. On a different approach, Olivry et al. [7] evaluating the recognition of several hydrolyzed poultry extracts on sera from dogs and cats with elevated chicken-specific serum IgE, noted that only extensively hydrolyzed poultry feather prevented the IgE recognition by poultry-specific IgE. Though the most known food allergens come from animal sources of protein [1], some grains may also have potential protein allergens, triggering an IgE-mediated response in dogs and cats [31]. Reports by Olivry and Bexley [32] show that serum from corn-sensitized dogs and cats did not have measurable IgE against proteins isolated from cornstarch, while kernel and flour extracts promoted IgE recognition. Therefore, the combination of hydrolyzed proteins and carbohydrate sources with limited protein content and proteome are highly recommended for hypoallergenic diets.

The 16S rRNA gene sequencing did not show differences in fecal communities between dogs fed hydrolyzed or common protein source diet. Although no differences were observed for fecal bacterial phyla between groups, the relative abundance verified for the HCLP group is in accordance with a previous study by Martínez-López et al. [33], in which healthy adult dogs also fed a hydrolyzed chicken liver diet for 6 weeks showed Firmicutes (median of 55%), Fusobacteria (median of 17%) and Bacteroidetes (median of 16%) as the most abundant bacterial phyla. Similarly, Bresciani et al. [34] did not find changes on fecal microbiota of healthy dogs after 60 days of an animal protein-free diet consumption. Interestingly, the fecal microbiota of healthy dogs is co-dominated by these three phyla: Firmicutes, Fusobacteria and Bacteroidetes [35, 36].

*Fusobacterium*, the most predominant genera in both groups, is associated with health in dogs [37] and can be used as a therapeutic marker for selected ingredients since it is reported to be reduced in dogs with gastrointestinal diseases [38]. Also, some *Fusobacterium* species are associated with SCFA synthesis from protein sources [39].

Although we did not evaluate end-fermentation products, a higher abundance of genera related to SCFA production were verified. Followed by *Fusobacterium*, dogs fed both dietary treatments showed a high abundance of *Bacteroides*, [*Prevotella*] and *Blautia*, genera associated with SCFA production. Among SCFA, butyrate is widely recognized by its preferred role as energy source for colonocytes, thus improving gut health [40].

Alpha diversity was not affected by dietary treatments, but PCoA plots based on Bray-Curtis and D_0.5 UniFrac distance metrics showed a clear separation between control group and HCLP group, which was also verified previously in dogs fed hydrolyzed and anallergenic diets [33, 41].

Additionally, the 16S rRNA sequencing evidenced the pronounced variability among dogs within the same dietary treatment in both phyla and genera relative composition, as previously described by Garcia-Mazcorro et al. [42]. The authors also found that the microbiome is relatively stable after a period of 2 weeks, so our feeding program was long enough to stabilize the gut microbiome.

In contrast to different types of fibers widely recognized for their prebiotic impact on microbiota composition and fermentative end-products related to gut health [43–45], some authors suggest that hydrolyzed diets affect the canine microbiome on a functional level instead of a taxonomic [33]. Perhaps a metabolomic approach could be more specific for identifying possible modifications in the HCLP group.

In general, the microbiome composition of dogs tends to be more affected by macronutrient modifications than by the ingredient itself. A recent study verified that dogs fed diets with similar macronutrient contents, one based on vegetable proteins and the other with mixed animal and vegetable proteins, did not showed differences in the microbiome composition [34]. This study could explain the absence of significant differences in our study, as both control and HCLP diet were formulated to be isonutritive and the main difference was the animal protein ingredient.

Overall, the present study showed that a diet based on HCLP was able to maintain all hematological markers within the reference intervals for adult dogs. Furthermore, 45 days of feeding with both diets decreased the concentrations of IL-4, IL-6, IL-7, IgA and IgE, cytokines and immunoglobulins related to the allergic response. Future studies are needed to evaluate different levels of the hydrolyzed protein and, more specifically, the end fermentation products that could be improved by this ingredient, since a higher abundance of specific genera related to SCFA production was verified in HCLP dogs.

## Supporting information

S1 File Full data set. Control, poultry by-product + bovine meat and bone meals-based diet; HCLP, hydrolyzed chicken liver powder-based diet.
(XLSX)
